# Highly Ordered DNA Framework Interface Enables Efficient Enzymatic Oligonucleotide Synthesis

**DOI:** 10.1002/advs.202505868

**Published:** 2025-09-03

**Authors:** Kunjie Li, Dongbao Tang, Xiaoyun Lu, Xinyao Yang, Luxuan Liu, Zhaoyuan Jia, Zhi Zhu, Yuyu Tan, Huimin Zhang, Chaoyong Yang

**Affiliations:** ^1^ The Ultra‐fast/Micro‐nano Technology and Advanced Laser Manufacturing Key Laboratory of Hunan Province College of Electrical Engineering University of South China Hunan 421001 China; ^2^ The MOE Key Laboratory of Spectrochemical Analysis and Instrumentation State Key Laboratory of Physical Chemistry of Solid Surfaces Department of Chemical Biology College of Chemistry and Chemical Engineering Innovation Laboratory for Sciences and Technologies of Energy Materials of Fujian Province (IKKEM) Xiamen University Xiamen 361005 China; ^3^ Zhonghe Gene Technology Co., Ltd Tianjin 300308 China

**Keywords:** DNA information storage, DNA synthesis efficiency, synthetic biology, tetrahedral DNA nanostructure

## Abstract

*De novo* DNA synthesis plays crucial roles in life science. Enzymatic oligonucleotide synthesis (EOS) has attracted interest due to longer synthesized chains, simple procedure, cost‐effectiveness, and environmental friendliness. However, unlike chemical synthesis dominated by small molecule, the EOS relies on enzyme reacting with primers. It remains challenging due to restricted accessibility caused by the anisotropy of initiator primers and the spatial hindrance of enzymes. Herein, this study developes a nanoscopic interface functioned with 3D DNA framework to achieve efficient EOS. The highly ordered DNA framework – tetrahedral DNA nanostructures (TDN) provide an ordered upright orientation and reasonable spacing for primers to enhance enzyme accessibility. Compared to single‐stranded structures, the TDN scaffold significantly enhances the enzyme's substrate affinity and catalytic reaction kinetics. As for the synthesis of five given patterned sequences, TDN scaffold effectively reduces the occurrence of deletion errors with increasing yield. Finally, efficient TDN‐based EOS is employed for DNA information storage by synthesizing a 60‐nucletide DNA fragment with a stepwise yield of 96.82%, allowing the accurate retrieval of 15 bytes of text information. The TDN‐based EOS paves the way for developing more efficient and accurate DNA synthesis methods, laying a robust foundation for future applications in DNA storage and genetic research.

## Graphical abstract

Tetrahedral DNA nanostructures (TDN) ‐based nanoscopic interface provides ordered upright orientation and optimal spacing for initiator primers, enhancing enzyme accessibility in interfacial enzymatic oligonucleotide synthesis (EOS). Notably, efficient TDN‐based EOS is employed for DNA information storage by synthesizing a 60‐nucletide DNA fragment, allowing the accurate retrieval of 15 bytes of text information.

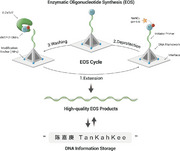

## 1 Introduction

*De novo* DNA synthesis acts essential roles in the vast majority of biological fields.^[^ ^1^ ^]^ Currently, DNA synthesis relies on the well‐established phosphoramidite method, initially proposed by Beaucage and Caruthers in 1981.^[^ ^2^ ^]^ However, due to the natural limitations of chemical synthesis, such as intricate procedures, high costs, production of hazardous waste, and limited length, which impede its utilization in cutting‐edge applications such as whole genome synthesis, DNA origami, and information storage.^[^ ^3^, ^4^, ^5^, ^6^ ^]^

Enzymatic oligonucleotide synthesis (EOS) is an emerging approach for *de novo* production of high‐quality DNA with controllable length.^[^ ^7^ ^]^ Represented by terminal deoxynucleotidyl transferase (TdT) and its engineered variants, EOS offers the following advantages: 1) Operating under mild aqueous conditions, EOS minimizes byproduct formation and DNA damage, allowing for the synthesis of longer oligonucleotides (oligo);^[^ ^8^ ^]^ 2) Featuring a simple procedure with two‐step extension, EOS not only shortens the synthesis cycle but also achieves cost reductions by several orders of magnitude;^[^ ^9^ ^]^ 3) EOS is environmentally friendly as it does not use hazardous organic chemicals or produce toxic waste.^[^ ^10^ ^]^ EOS relies on the combination of solid‐phase synthesis and enzymatic polymerization of nucleotides. Temporarily blocked nucleotide building blocks by 3′‐*O*‐masking groups, can be incorporated onto a solid support bound primer by template‐independent polymerases. After removal of excess polymerase and building blocks, the masking groups are removed, and the extended primer can be subjected to subsequent synthetic cycles.^[^ ^11^ ^]^ To enhance the efficiency of controllable DNA synthesis with single‐base resolution, mutant TdT variants have been screened and engineered for the incorporation of 3′‐temporarily blocked nucleotides. Notably, in our previous study, the engineered *Zonotrichia albicollis* TdT (E‐ZaTdT) demonstrated superior performance compared to numerous other variants, achieving highly efficient and precise incorporation of 3′‐ONH_2_‐dNTPs.^[^ ^12^, ^13^ ^]^ Although the modified enzymes have significantly enhanced the catalytic capability of temporarily blocked nucleotides, this strategy still leads to notable synthesis errors compared to chemical synthesis and wild‐type enzymatic synthesis methods, primarily deletion errors.^[^ ^8^, ^12^, ^13^ ^]^ Furthermore, unlike the chemical synthesis dominated by small molecule, the EOS requires bulky enzyme binding on primer for the extension reaction to take place. These errors are further amplified at the solid‐liquid interface (where high‐throughput EOS matters) ^[^ ^10^, ^14^ ^]^ due to the restricted accessibility caused by the anisotropy of the initiator primers (including molecular aggregation, inter‐strand interaction, and nonspecific adsorption) and the spatial hindrance of transferase.^[^ ^15^, ^16^ ^]^ In oligo synthesis, such errors are unacceptable because the gradual accumulation of minor differences will be exponentially magnified by steps, ultimately resulting in the inability to produce long‐chain DNA.

**Figure 1:**
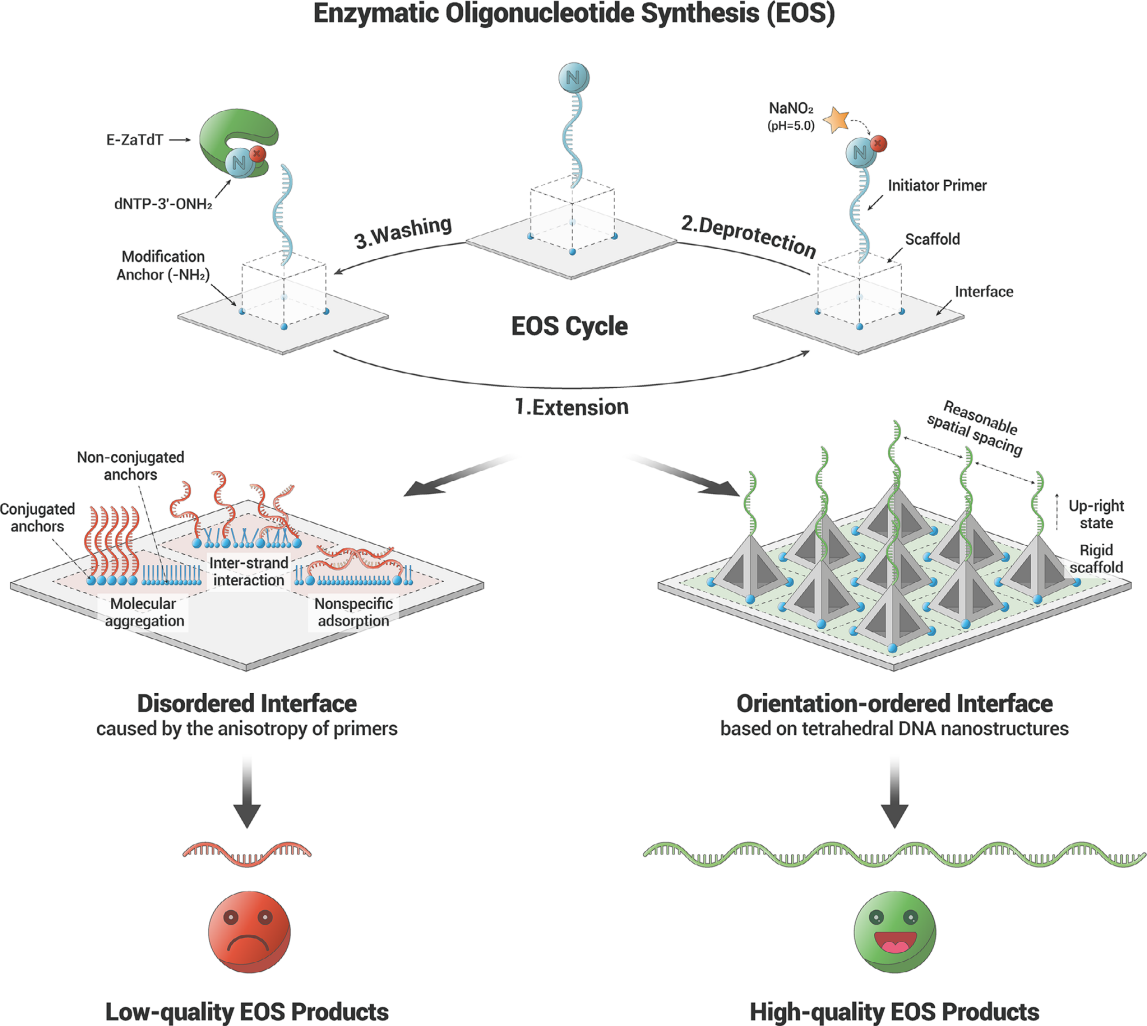
Highly ordered DNA framework interface for high‐quality EOS. The restricted accessibility caused by the anisotropy of initiator primers and the spatial hindrance of enzymes inevitably reduce the quality of EOS products. TDN sever as enclosed and rigid scaffolds for the initiator primers, maintaining reasonable spatial spacing and an up‐right orientation for efficient and accurate enzymatic reaction. In the disordered interface, potential scenarios of random modifications are depicted, including molecular aggregation, inter‐strand interactions, and nonspecific adsorption.

To tackle this issue, we introduce a nanoscopic interface functionalized with well‐defined three‐dimensional DNA framework to improve the spatial arrangement of initiator primers and their interaction with enzyme. Owing to the precise and programmable nucleotide base pairing (A–T and G–C), DNA framework provides a convenient approach to controlling biomolecule‐confined surfaces.^[^ ^17^, ^18^ ^]^ Tetrahedral DNA nanostructures (TDN) as an elegant example, have been proven to serve as a rigid framework that enhances the spatial arrangement and upright orientation of molecules anchored on interfaces, which has been demonstrated in various biological recognition applications, including nucleic acids, proteins, small molecules, and cells.^[^ ^19^, ^20^, ^21^ ^]^ Similarly, TDN have been employed to achieve spatially ordered arrangements of enzymes or DNA under highly control in applications involving enzymatic reactions at interfaces.^[^ ^22^, ^23^, ^24^ ^]^ While these studies have demonstrated the utility of TDN in improving diverse biomolecular interactions, the application in enhancing biological manufacturing efficiency—particularly in EOS—remains underexplored. Therefore, we developed a nanoscopic interface engineering strategy based on TDN to achieve upright‐ordered spatial arrangement of initiator primers, aiming to enhance the kinetics of enzymatic incorporation of temporarily blocked nucleotides on the interface (**Figure** **1**). With the rigid structure of TDN, the primers can be arranged on the solid‐phase interface with upright‐orientated nano‐topography to avoid molecular aggregation or non‐specific adsorption. Meanwhile, confined tetrahedral nanostructure scaffolds also reduce the local overcrowding effect with a reasonable spatial spacing on the interface, making enzyme accessible to primers and leading to an efficient reaction and accurate elongation.

## 2 Results

### 2.1 Assembly and Characterization of Initiator‐TDN on Interface

Specifically, we utilized well‐defined TDN, each with edges composed of 17 base pairs (corresponding to a length of 5.8 nm), to fabricate highly ordered DNA framework interfaces for efficient EOS. These enclosed tetrahedral nanostructures, with their precisely controlled spatial arrangement on the interface (quantitatively characterized as ≈5.2 nm spacing based on the prior work of M. Lin et al. ^[^ ^25^ ^]^), mitigate issues such as molecular aggregation and inter‐strand entanglement caused by excessive crowding of initiator primers. Additionally, the rigid scaffold of TDN allows the initiator primers to maintain an up‐right oriented nanostructure on the interface, facilitating the efficient incorporation of dNTP‐3′‐ONH_2_ by E‐ZaTdT. Therefore, this strategy offers a convenient and efficient approach to finely control the interfacial spatial arrangement of initiator primers at the nanoscale, thereby improving the kinetics and accuracy of enzymatic reaction for longer and accurate oligo synthesis.

**Figure 2:**
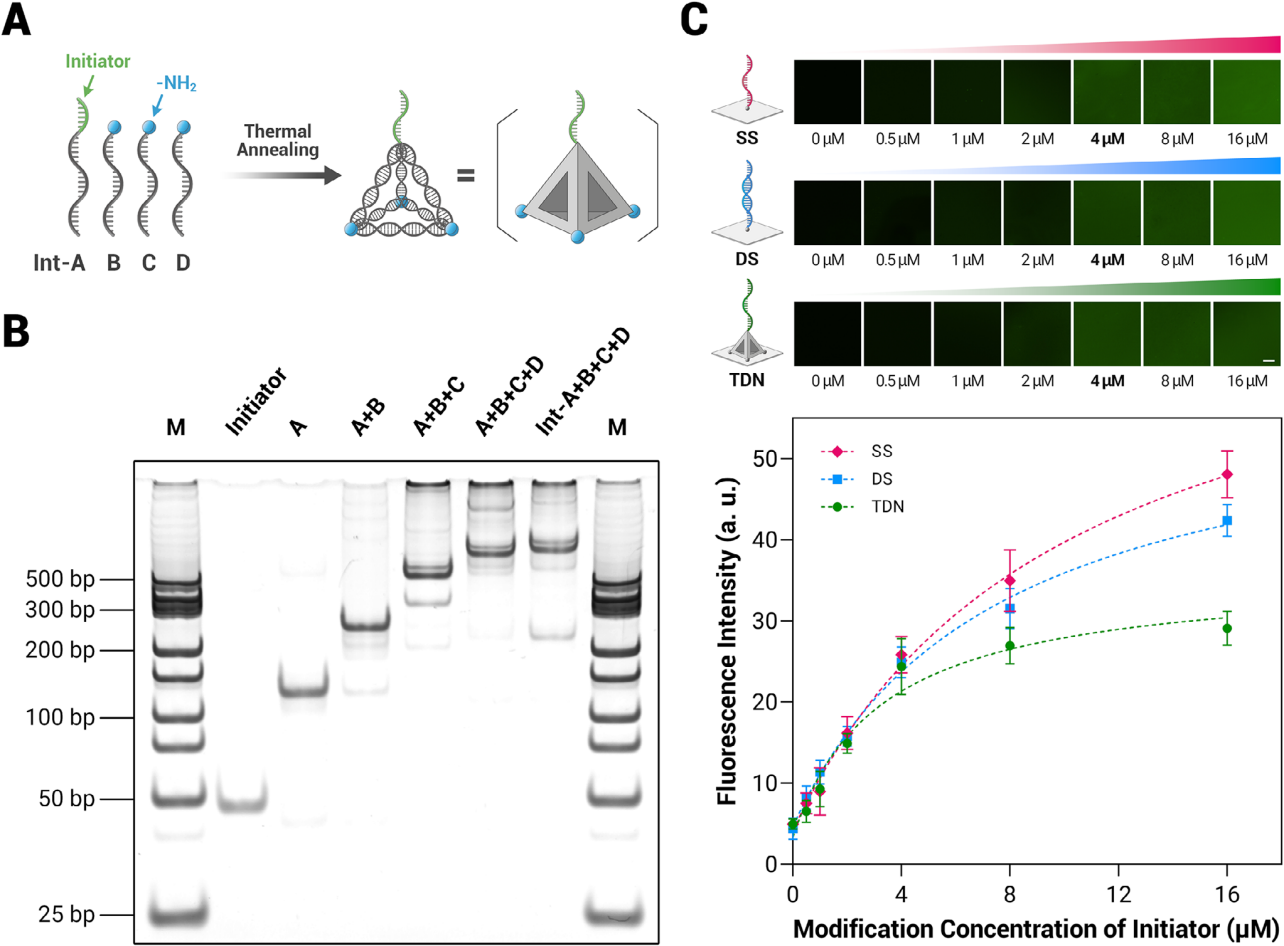
Characterization of Initiator‐TDN annealing assembly and interfacial modification. A) Schematic illustration of the Initiator‐TDN self‐assembly. B) Native PAGE (8%) characterization of the progressive self‐assembly of Initiator‐TDN. C) Representative fluorescence images (upper) and corresponding fluorescence quantification (bottom) of three different types of modifying the initiator primers on the PDMS interface, respectively. Int: initiator, SS: single‐stranded DNA, DS: double‐stranded DNA, TDN: tetrahedral DNA nanostructure. Scale bar = 100 µm. Data are calculated from *n* = 3 independent experiments and presented as mean ± SD.

The TDN can be self‐assembled from four 55‐nucleotide (nt) strands, A, B, C, and D, which are partially complementary to each other. To introduce an initiator primer at the top of the TDN, we incorporated an Int‐A strand, which includes an 18‐nt initiator and the A strand. The Initiator‐TDN was one‐step assembled from Int‐A, B, C, and D strands via thermal annealing (**Figure** **2**A). As shown in Figure 2B, the progressive formation of Initiator‐TDN was characterized by native polyacrylamide gel electrophoresis (PAGE). The migration distance of the assembled product increased with the sequential addition of each DNA strand, confirming the stepwise assembly of the Initiator‐TDN. ^[^ ^19^, ^21^ ^]^ While the migration position of the TDN does not perfectly align with the molecular weight ladder due to factors such as its three‐dimensional conformation, shape, charge distribution, and flexibility, this discrepancy does not hinder the evaluation of successful self‐assembly. The primary band observed in each lane corresponds to the increased size of the intermediate or final structure, indicating the successful formation of the Initiator‐TDN at each assembly step. Based on image analysis of band intensity, the yield of the Initiator‐TDN was calculated to be over 80%. As a typical material for high‐throughput microfluidics, polydimethylsiloxane (PDMS) was selected as the reaction interface for EOS in this study. The 3′ ends of the B, C, and D strands were modified with amino groups, serving as modification anchors. We employed a 3‐mercaptopropyltrimethoxysilane (MPTS)/n‐γ‐maleimidobutyryl‐oxysuccinimide ester (GMBS) method to covalently bond the amino‐modified DNA strands to the PDMS surface (Figure S1, Supporting Information). To compare the effects of different interface modification methods on EOS efficiency in subsequent experiments, we used three types of DNA structure to anchor the initiator primers to the PDMS interface: single‐stranded scaffold (SS), double‐stranded scaffold (DS), and TDN scaffold. As shown in Figure 2C, successful modification was confirmed by fluorescence imaging. Quantitative fluorescence analysis indicated that at a modification concentration of 4 µm, the amount of initiator attached to the interface was similar across the three types, providing consistent reaction sites for subsequent enzymatic kinetics comparisons (Figure S2, Supporting Information). Therefore, 4 µm was selected as the modification concentration for the initiator primers in following experiments.

### 2.2 Quantitative Assay for Evaluating EOS Efficiency

To quantitatively analyze the efficiency of EOS, we employed a pyrophosphate ion (PPi)‐based bioluminescence assay to evaluate the efficiency of E‐ZaTdT in incorporating dNTP‐3′‐ONH_2_. Similar to pyrosequencing, a sequencing‐by‐synthesis method via a cascade of four enzymatic reactions, we recorded the bioluminescence signal generated from PPi by‐products produced during the extension of the oligo by E‐ZaTdT (Figure S3A, Supporting Information). As shown in Figure S3B (Supporting Information), the luminescence intensity exhibited a linear relationship with PPi concentrations ranging from 1 to 10 µm (*R* ^2^ = 0.9956), favoring the quantitative requirement for synthesis efficiency analysis. To assess the accuracy of this method, we tested the recoveries of low (1.88 µm), medium (3.75 µm), and high (7.50 µm) spiked concentrations within the linear range (Figure S3C, Supporting Information). The results demonstrated excellent recoveries ranging from 96.7% to 102.1%. The assay was first employed to characterize that TDN scaffold introduces minor nonspecific confounding effects, demonstrating negligible impact on the quantitative evaluation of EOS efficiency (Figure S4, Supporting Information). Subsequently, we used this PPi‐based assay to optimize the reaction conditions for EOS on interface, including reaction temperature, cobalt ion concentration, and surfactant concentration (Figure S5A–C, Supporting Information). Finally, we determined the optimal conditions for EOS to be a reaction temperature of 40 °C, a cobalt ion concentration of 0.63 mm, and a Triton X‐100 concentration of 0.01% (v/v).

### 2.3 Comparison of EOS Kinetics on Different Interfacial Scaffolds

**Figure 3:**
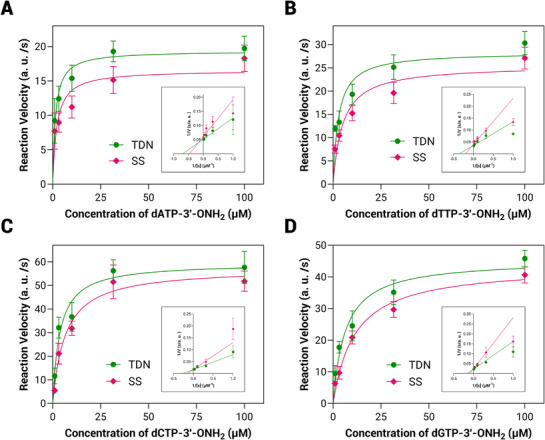
Comparison of the EOS kinetics for TDN and SS scaffolds. The substrate includes A) dATP‐3′‐ONH_2_, (B) dTTP‐3′‐ONH_2_, C) dCTP‐3′‐ONH_2_ and D) dGTP‐3′‐ONH_2_. The bottom right panel shows the Lineweaver‐Burk plots. [S]: concentration of substrate, V: reaction velocity. Data are calculated from *n* = 3 independent experiments and presented as mean ± SD.

Michaelis–Menten model was used to evaluate the impact of SS, DS and TDN scaffolds on EOS kinetics (**Figure** **3**; Figure S6A, Supporting Information). By measuring the reaction velocity (V, a. u./s, with Δ changes calculated from luminescence intensity) of E‐ZaTdT incorporating dNTP‐3′‐ONH_2_ (A, G, C, T) at substrate concentrations ([S], µm) of 1, 3.16, 10, 31.6, and 100 µm, the enzyme kinetic curves were generated and converted them into Lineweaver‐Burk plots. A comparison of enzyme kinetic curves between the SS, DS, and TDN scaffolds confirmed that TDN exhibited improved EOS kinetics. Using Lineweaver‐Burk plots, we determined the kinetic parameters (*K_m_*, *k_cat_*, and *k_cat_*/*K_m_*) of these two interfaces types for quantitative comparison (Table 1). Using the thymine as an example, the *K_m_* value of EOS with the TDN scaffold was 42.94% lower than that with SS, indicating a significant enhancement of the enzyme's substrate affinity at the interface by the TDN scaffold. More importantly, the *k_cat_* and *k_cat_*/*K_m_* values of EOS with TDN modification were 10.89% and 94.51% higher, respectively, than those with SS, demonstrating that the TDN scaffold substantially improves the enzyme's substrate conversion rate and catalytic efficiency at the interface. Similar enhancements were observed for the other three bases, as detailed in Table 1. Although the DS scaffold offers a degree of rigidity that improves enzyme‐substrate interactions by reducing steric hindrance, its substrate affinity and catalytic efficiency remain lower than those of the TDN scaffold (Figure S6B,C, Supporting Information).

**Table 1:** Kinetic parameters (*K*_m_, *k*_cat_, *k*_cat_/*K*_m_) of three different interfacial modification modes for the single‐nucleotide incorporation reaction.

Kinetic parameters	Interfacial modification Mode	Type of dNTP‐3′‐ONH_2_			
		A	T	C	G
*K*_m_ [µM]	SS	2.17	4.96	6.49	11.29
	TDN	1.44	2.83	3.73	6.34
Parameter Gain		33.64%	42.94%	42.53%	43.84%
*k*_cat_ [s^−1^]	SS	2.92	4.50	10.10	7.67
	TDN	3.41	4.99	10.48	8.00
Parameter Gain		16.78%	10.89%	3.76%	4.30%
*k*_cat_/*K*_m_ [µM^−1^ s^−1^]	SS	1.35	0.91	1.56	0.68
	TDN	2.36	1.77	2.81	1.26
Parameter Gain		74.81%	94.51%	80.13%	85.29%

To further characterize enzyme‐primer interactions, we employed E‐ZaTdT to sequentially incorporate dCTP‐Cy3 and dCTP‐Cy5, enabling quantitative fluorescence resonance energy transfer (FRET) efficiency measurements (Figure S7, Supporting Information). We separately measured the FRET efficiency of the three interfacial frameworks at 1 min (representing initial reaction kinetics) and 10 min (reflecting final product yield). The results demonstrate that TDN scaffolds exhibit significantly higher FRET efficiency (58.68% ± 6.94%) compared to both SS (28.33% ± 5.50%) and DS (36.59% ± 4.58%) scaffolds at the 1 min time point, indicating enhanced initial enzymatic kinetics. This kinetic advantage persisted at the 10 min measurement, with TDN scaffolds achieving 62.84% ± 7.65% FRET efficiency, significantly outperforming both control scaffolds. Taken together, the TDN framework exhibits superior enzymatic reaction kinetics for interfacial EOS.

### 2.4 Enhanced Efficiency of Interfacial EOS via TDN Scaffold

Subsequently, we assessed the enzymatic synthesis yield of a 9‐base DNA fragment (5′‐GCTGCTGCT‐3′) across three distinct interface modification modes (Figure S8A, Supporting Information). In the 9‐cycle synthesis, the stepwise yield of EOS on the TDN scaffold achieved 96.97%, resulting in a full‐length yield of 75.83% (Figure S8B,C, Supporting Information), markedly higher than the 93.89% stepwise yield (full‐length yield = 56.85%) on the SS scaffold and the 93.93% stepwise yield (full‐length yield = 57.09%) on the DS scaffold. Deletion errors are the most prevalent type of synthesis error in EOS, primarily resulting from the enzyme's inaccessibility to the initiator primers. ^[^ ^8^, ^12^, ^13^ ^]^ Compared to the SS and DS scaffolds, the TDN‐based interface modification significantly reduced the proportion of deletion errors (from 8.47% for SS and 7.26% for DS to 3.05% for TDN, Figure S8D, Supporting Information). These improvements may attribute to the ordered orientation of the primers on the interface, enhancing their accessibility to the enzyme.

**Figure 4:**
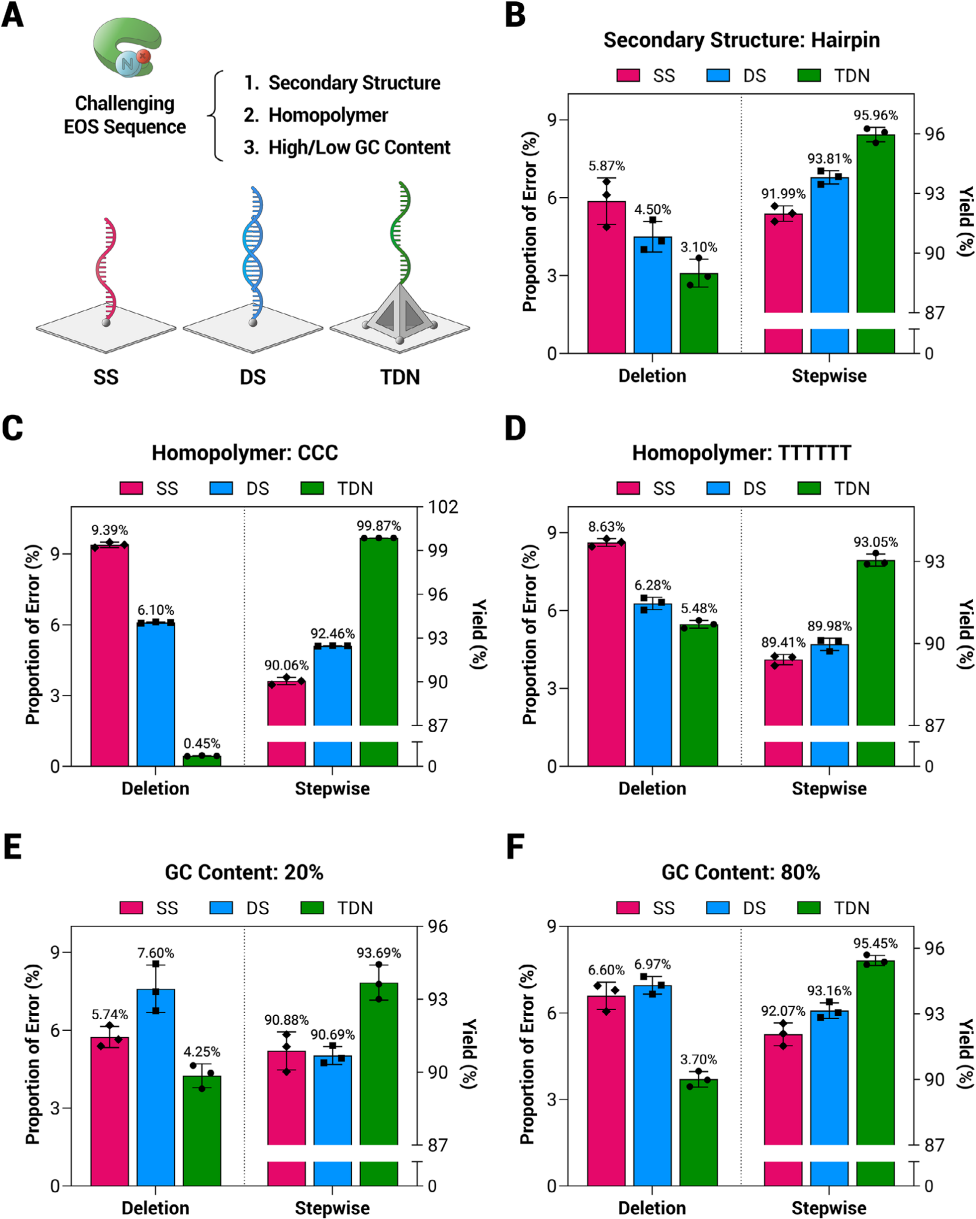
Comparison of the stepwise yield and deletion rate of EOS for five challenging sequence patterns on SS, DS, and TDN scaffolds. A) Schematic representation of initiator primers for EOS on SS, DS, and TDN scaffolds. The five challenging sequence patterns include: B) a hairpin structure of free energy ΔG = −7.10, C) homopolymers of 5′‐CCC‐3′, (D) homopolymers of 5′‐TTTTTT‐3′, E) low GC content of 20%, and F) high GC content of 80%. The comparative parameters are proportion of deletion errors (left) and stepwise yield (right). Data are calculated from *n* = 3 independent experiments and presented as mean ± SD.

In advanced applications of *de novo* DNA synthesis, the sequence demands are highly diverse. ^[^ ^26^, ^27^ ^]^ Specific sequence patterns present significant challenges for synthesis, including those with stable secondary structures (e.g., hairpins), single‐nucleotide repeats (e.g., homopolymers), and GC content beyond the range of 40–60%. These sequences are notoriously difficult to synthesize with higher error rates.^[^ ^28^ ^]^ To demonstrate the superiority of the TDN‐based orientation‐ordered interface for EOS of challenging sequences, we selected five reported sequence patterns for comparison with SS, DS and TDN scaffolds (**Figure** **4**A). These include a hairpin structure (free energy ΔG = −7.10: 5′‐CGAGCTAGTCAGCTCG‐3′), two homopolymer sequences (5′‐CCC‐3′ and 5′‐TTTTTT‐3′), and two sequences with low and high GC content (20%: 5′‐ATATGATATC‐3′ and 80%: 5′‐CGCGTCGCGA‐3′). In the EOS of these five sequences, the TDN‐based interface consistently exhibited superior stepwise yields and lower deletion error rates, indicating the orientation‐ordered interface significantly enhances the accuracy of EOS for sequences that are difficult to synthesize (Figure 4B‐F). Notably, in the synthesis of the homopolymer sequence 5′‐CCC‐3′, the deletion rate at TDN‐based interface was only 0.45%, representing reductions of 8.94% and 5.65% compared to SS and DS, respectively (Figure 4C). Based on the quantitative result of next‐generation sequencing (NGS), this improvement resulted in a stepwise yield of 99.87% (=(0.9961)^1/3^) for TDN‐based EOS, significantly surpassing the 95.89% (=(0.8818)^1/3^) yield reported for homogeneous EOS by S. Palluk et al.^[^ ^8^ ^]^ Therefore, the TDN‐based orientation‐ordered interface markedly improve the accuracy of EOS at solid interface for difficult‐to‐synthesize sequences, promising high‐fidelity and unbiased synthetic oligo for applications such as barcoding for spatial transcriptomics and DNA‐based information storage.

### 2.5 TDN‐Based EOS for DNA Information Storage

**Figure 5:**
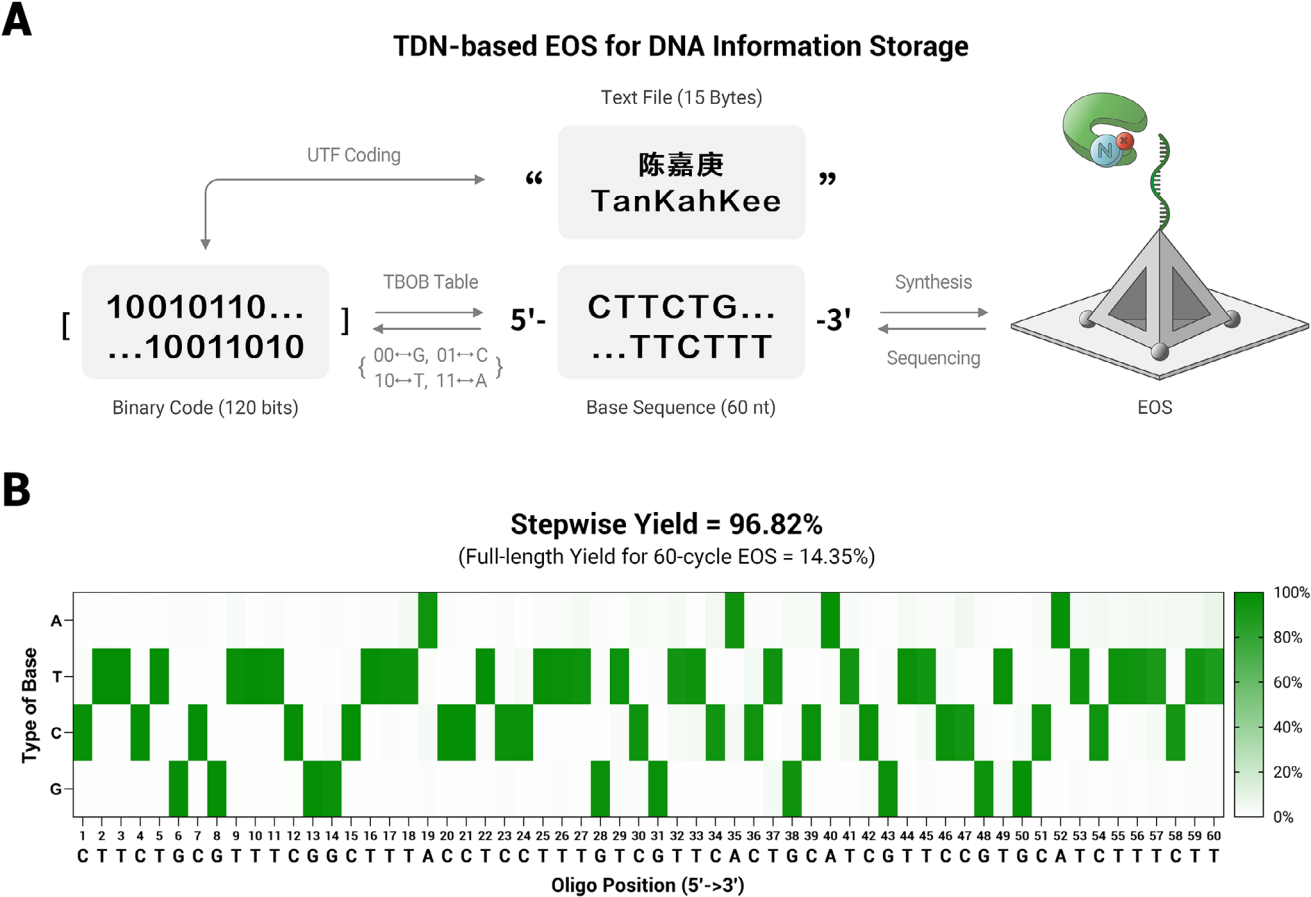
TDN‐based EOS for DNA information storage. A) Schematic representation of DNA data storage by TDN‐based iEOS. UTF: Unicode Transformation Format. TBOB: Two‐Bits‐to‐One‐Base. B) Yield evaluation and information readout via NGS of synthesized 60‐nt oligo. The heatmap was plotted by traversing NGS results position by position from the initiator (i.e., after determining the previous base to screen the sequences, the current base was read based on the most frequent base type at each position).

DNA is emerging as a compelling candidate for next‐generation information storage media, owing to its superior storage density, extended preservation time, and lower power consumption. ^[^ ^29^, ^30^ ^]^ A critical step in DNA information storage is the synthesis of long DNA sequences to enhance storage capacity. Employing EOS for data writing is increasingly favored due to its potential for extended synthesis lengths, low cost, and environmental friendliness.^[^ ^4^, ^31^ ^]^ Meanwhile, the microfluidic interface serves as a fundamental platform for achieving high‐density and high‐capacity DNA information storage.^[^ ^16^ ^]^ Therefore, efficiently synthesizing long DNA sequences using EOS at the interface is essential, though it remains a significant challenge.^[^ ^31^ ^]^ Here, we employed efficient TDN‐based EOS for DNA information storage (**Figure** **5**A). A text file representing the name of Tan Kah Kee, the founder of Xiamen University, containing three Chinese characters and nine English letters (totaling 15 bytes), was converted into a 120‐bit binary code using Unicode Transformation Format (UTF) encoding. The resulting 120‐bit binary data was encoded into a 60‐nt DNA fragment (5′‐CTTCTGCGTTTCGGCTTTACCTCCTTTGTCGTTCACTGCATCGTTCCGTGCATCTTTCTT‐3′) by translating 00, 01, 10, and 11 to G, T, C, and A, respectively. Multi‐cycle EOS was performed on the TDN‐based orientation‐ordered interface to achieve data writing. The synthesized oligo were then subjected to NGS to retrieve the stored information. NGS results indicated a stepwise yield of 96.82% over 60 cycles of EOS and a full‐length yield of 14.35%. By employing a majority vote strategy, the target sequence was successfully identified and decoded to retrieve the stored 15 bytes text file (Figure S9, Supporting Information).^[^ ^32^ ^]^ Furthermore, the NGS results were traversed position by position from the initiator primer and the stored information was retrieved accurately (Figure 5B; Figure S10, Supporting Information). To confirm the stability of TDN scaffolds during EOS—critical for long DNA synthesis—we performed PAGE and FRET characterization.^[^ ^19^ ^]^ Native PAGE (8%) revealed identical band positions without smearing or tailing, confirming maintained structural integrity after synthesis (Figure S11A, Supporting Information). FRET analysis showed consistent efficiency (28.6% ± 2.5% pre‐synthesis versus 27.4% ± 3.1% post‐synthesis), proving preserved inter‐vertex distances and structural geometry under EOS conditions (Figure S11B, Supporting Information). Taken together, these results demonstrate that TDN‐based EOS can be employed for high‐accuracy and low‐redundancy DNA information storage.

## 3 Discussion

Oligo synthesis is a cornerstone technique in the fields of life science and synthetic biology, playing a central role in a range of cutting‐edge applications such as DNA‐based information storage, artificial life, precision medicine, drug development, and green manufacturing. ^[^ ^4^, ^33^ ^]^ As synthetic DNA becomes increasingly integral to biological research, the development of more efficient, controllable, and scalable DNA synthesis methods is critical. EOS has garnered significant attention in recent years due to its potential to overcome the limitations of traditional chemical methods, such as short chain lengths, high costs, and organic solvent pollution. However, EOS faces challenges related to the accessibility of both the enzyme and the initiator primers, which leads to low enzymatic reaction efficiency. This issue is further exacerbated at solid‐liquid interfaces, where it can significantly hinder the achievement of high‐throughput EOS. Therefore, enhancing the accessibility of the enzyme and initiator primers at the interface becomes a critical challenge that is inevitable for EOS to replace phosphoramidite chemistry as the next‐generation DNA synthesis method.

In this context, the TDN‐enhanced interfacial EOS strategy presents a promising solution, as demonstrated by our experimental results. However, its translation to practical applications depends critically on resolving key challenges in large‐scale TDN production, particularly regarding cost efficiency, yield optimization, and purity control. First, the predominant cost in TDN production arises from the chemical synthesis of oligo chains required for assembly. Notably, these oligo do not require *de novo* sequence definition, which opens avenues for more economical production methods. Recent advances in bacteriophage‐DNAzyme systems have demonstrated scalable and cost‐efficient production of ssDNA with single‐base precision, offering a viable alternative for DNA nanostructure fabrication. ^[^ ^34^, ^35^ ^]^ This biotechnological approach achieves a three‐order‐of‐magnitude cost reduction relative to conventional chemical methods, enhancing the feasibility of large‐scale TDN implementation. Moreover, current TDN assembly protocols consistently achieve yields of 85–90% at the laboratory scale.^[^ ^19^, ^21^ ^]^ Through optimized purification techniques such as ultrafiltration or engineered magnetic probes, purity levels up to 96.8% can be obtained.^[^ ^36^, ^37^ ^]^ The integration of these methods with industrial‐scale separation and analysis platforms (e.g., high‐performance liquid chromatography) demonstrates strong potential for scalable TDN manufacturing. These advancements in yield and purity are critical for ensuring the reproducibility and efficiency of TDN‐enhanced EOS, particularly in high‐throughput applications.

## 4 Conclusion

Herein, we developed an orientation‐ordered initiator‐modified interface using TDN scaffolds to achieve efficient and accurate EOS. The rigid TDN framework confers a highly ordered upright orientation and controllable spacing to the initiator primers, enhancing enzyme‐primer accessibility. Enzymatic kinetics results show that the TDN scaffold significantly improves the enzyme's substrate affinity and catalytic efficiency on the interface. For the synthesis of challenging patterned sequences, TDN‐based EOS effectively reduces deletion error rates, thereby increasing oligo yields. Finally, efficient TDN‐based EOS was utilized for DNA information storage by synthesizing a 60‐nt DNA fragment with a stepwise yield of 96.82%, enabling accurate retrieval of 15 bytes of text information. PDMS is a widely used interface material for high‐throughput microfluidic chips and is employed here as a representative example to validate our method. We anticipate that this strategy can be further refined to engineer optimal interfaces for high‐throughput EOS. Additionally, this approach serves as an effective demonstration and presents new opportunities for advancing DNA frameworks from conventional biorecognition applications to the practical demands of biological manufacturing.

## 5 Experimental Section

### Assembly and Characterization of Initiator‐TDN

The assembly of the TDN followed the well‐established protocol.^[^ ^20^ ^]^ All oligo) were synthesized and purified using high‐performance liquid chromatography (HPLC) by Sangon Biotechnology, with sequences detailed in Table S1 (Supporting Information). The four oligo strands for constructing Initiator‐TDN were quantified for their molar concentration by measuring the optical density at 260 nm using a NanoDrop Spectrophotometer (Thermo Scientific). For the assembly of Initiator‐TDN, equimolar amounts of DNA strands (Int‐A, B, C, and D) were dissolved in TM buffer (20 mm Tris, 50 mm MgCl_2_, pH 8.0), heated to 95 °C for 10 min, and then cooled to 4 °C for 20 min. The assembled product was characterized by native polyacrylamide gel electrophoresis (PAGE) using 8% acrylamide. The formation yield was analyzed from the PAGE image using ImageJ software by comparing the band intensities.

### Modification of Initiator Primers on PDMS Interface

To construct a PDMS (Sylgard 184, Dow Corning) interface, 100 µL of pre‐mixed prepolymer (w/w = 10:1) was added to a 96‐well plate or tube and heated at 100 °C for 15 min. The PDMS interface was activated by oxygen plasma for 1 min, followed by incubation with 5% (v/v) (3‐mercaptopropyl)trimethoxysilane (MPTS, Sigma–Aldrich) in anhydrous ethanol for 1 h to achieve a thiolated surface. After ethanol washing, the interface was dried at 100 °C for 30 min. Subsequently, freshly prepared N‐γ‐maleimidobutyryl‐oxysuccinimide ester (GMBS, Sigma–Aldrich) (0.5 mg mL^−1^ in ethanol) was applied for 25 min to generate an NHS ester‐functionalized interface. Finally, amino‐modified scaffold strands in PBS buffer were incubated at room temperature for 1 h, followed by blocking of unreacted sites with 3% (m/v) BSA in PBS buffer. Initiator primers were modified on the PDMS interface in three modes: SS, DS and TDN (Table S1, Supporting Information), to compare the efficiency of enzymatic oligo synthesis in each mode. In SS mode, initiator primers were directly modified on the interface in a single‐strand state. In DS mode, initiator primers annealed with their amino‐modified complementary strands were modified on the interface in a double‐strand state. In TDN mode, initiator primers were modified on the interface in the assembled Initiator‐TDN state. To ensure consistent modification concentrations for the three modes, initiators modified with 6‐carboxyfluorescein at the 3′ end were used for quantitative analysis. Based on the input concentration of the initiator, concentrations of 0, 0.5, 1, 2, 4, 8, and 16 µm were tested for the three modification modes, respectively. The modified interface was imaged under a fluorescence microscope, and the images were quantitatively analyzed using ImageJ software.

### Interfacial EOS

The single‐nucleotide interfacial EOS on PDMS followed a well‐established three‐step protocol: extension, deprotection, and washing. ^[^ ^12^, ^13^ ^]^ Specifically, a 10 µL standard synthesis mixture was prepared by combining 0.5 mg mL^−1^ E‐ZaTdT, 0.25 mM 3′‐ONH_2_‐dNTPs (Firebird Biomolecular Sciences), 0.63 mm CoCl_2_, 100 mm NaCl, and 50 mm PBS buffer (pH 6.8). The reaction mixture was applied to the PDMS interface (well plate or tube), initiating the extension process at 40 °C for 10 min. The interfacial products were then deprotected for 1 minute using sodium nitrite buffer (0.7 m, pH 5, adjusted with nitrous acid) and washed twice with 50 mM PBS buffer (pH 6.8) in preparation for the next synthesis cycle. These three steps constitute a single‐base cycling process for synthesizing the desired DNA sequence.

### Bioluminescence Assay

To construct a linear standard curve, appropriate amounts of sodium pyrophosphate (Na_4_P_2_O_7_·10H_2_O) were added to Tris‐HCl buffer (20 mm, pH 8.5) to generate gradient concentrations of pyrophosphate ion (PPi) (0.5, 1.0, 1.5, 2.0, and 2.5 µm). The reaction buffer (100 mm Tris acetate, 0.5 mm EDTA, 5 mm magnesium acetate, and 0.01% v/v Tween 20, pH 7.6) was prepared at a 2× concentration, including 2 ng µL^−1^ ATP sulfurylase (7175‐AS, R&D Systems), 10 µm adenosine phosphosulfate (APS, A5508, Sigma–Aldrich), 0.24 µg/µL firefly luciferase (FL0001, MCLAB), and 1.2 µg µL^−1^ D‐luciferin (L9504, Sigma–Aldrich). Equal volumes of the PPi standard solution and the reaction buffer were mixed to initiate the bioluminescence reaction. After 1 min of reaction, the luminescence intensity was measured using a microplate reader (Tecan, Switzerland). To further investigate the accuracy of the assay, three concentrations of PPi (1.88, 3.75, and 7.50 µm) were added to the enzyme‐containing buffer, corresponding to low, medium, and high levels of enzymatic reaction. The recoveries for each level were tested. The measured luminescence intensity signals were substituted into the standard curve to obtain the detected concentrations, which were then divided by the actual concentrations to determine the recoveries for each level.

### FRET Analysis

Fluorophore‐labeled TDN (Cy3 donor; Cy5 acceptor) were immobilized on the functionalized interface. Fluorescence spectra were acquired using a microplate reader (Tecan, Switzerland) before and after the reaction, with excitation at 514 nm and emission collected from 540 to 750 nm. FRET efficiency (*E*) was calculated as:

(1) $$E=1-\left(I_{DA}/I_{D}\right)$$

where *I_DA_* and *I_D_* represent the maximum donor fluorescence intensity in the presence and absence of the acceptor, respectively. Three replicates were performed for each experimental condition, with background subtraction using buffer‐only measurements.

### Optimization of Interfacial EOS

The efficiency of EOS was affected by several experimental parameters, including the concentration of the surfactant Triton X‐100 (0%, 0.005%, 0.01%, and 0.02%, v/v), reaction temperature (20, 30, 40, and 50 °C), and cobalt ion concentration (0, 0.25, 0.63, and 1.25 µm). The reaction efficiency was assessed using a PPi‐based bioluminescence assay, with all conditions tested in triplicate.

### Enzyme Kinetics Analysis

Under the optimal conditions, the reaction system was prepared with 0.63 mm CoCl_2_, 100 mM NaCl, 0.01% Triton X‐100 (v/v), and 50 mm PBS buffer (pH 6.8) at 40 °C. For kinetic analysis of EOS, 3′‐ONH_2_‐dNTPs were used as substrates (S) at concentrations ranging from 1.0 to 100.0 µm. The reaction was initiated by adding E‐ZaTdT to a final concentration of 0.5 mg/mL and terminated by heating to 95 °C. Reaction progress was monitored by measuring PPi formation at various time points (0, 10, 20, 40, 80, and 160 s) using the previously described bioluminescence assay. Fluorescence intensity versus reaction time was plotted for each substrate concentration, and the slope of the initial linear phase was used to determine the reaction rate. The substrate concentration ([S]) and reaction rate (V) were then fitted to the Michaelis‐Menten equation and transformed into Lineweaver‐Burk plots to calculate the kinetic parameters (*K*_m_, *k*_cat_, and *k*_cat_/*K*_m_). Data fitting was performed using GraphPad Prism software. All conditions were tested in triplicate. To assess structural stability via FRET under standardized EOS conditions, sequential enzymatic incorporation of dCTP‐Cy3 (B8159, APExBIO) and dCTP‐Cy5 (B8161, APExBIO) was performed. The system first extended dCTP‐Cy3 for either 1 or 10 min, followed by a uniform 10 min dCTP‐Cy5 extension to complete FRET pair labeling.

### Evaluation of EOS by NGS

This section of the method was adapted from previously well‐established studies. ^[^ ^8^, ^12^, ^13^ ^]^ Specifically, next‐generation sequencing (NGS) was utilized to quantitatively evaluate the synthesized product. Following addition of the last cycle for each sequence, the 3′ hydroxyl termini of the synthesized products were first tailed with 250 µm polyA using 1 U/µL calf thymus TdT (M0315, NEB) as per manufacturer's instructions. Subsequently, a 50 mm dithiothreitol (DTT) solution in PBS (with 1% BSA) was added and incubated at 37 °C for 30 min to cleave the disulfide bonds at the terminus of the TDN‐A‐Int strand, facilitating the removal of synthesized oligo from the interface. Next, the sequencing library of resulting products was prepared for dual‐index paired‐end sequencing analysis in two PCR steps using Phanta super‐fidelity DNA polymerase (P505, Vazyme) as per manufacturer's instructions. In the first step, the polyA tailed products were amplified using universal primer with polyT (C1) and standard initiator (Int). Second, dual indices and Illumina sequencing adapters (P5 & P7) were further attached to the amplicons from the first‐step PCR. After amplification, each sample was purified by VAHTS DNA clean beads (N411, Vazyme) as per manufacturer's instructions and pooled in the same concentration for a shared PE150 run (NovaSeq 6000, Illumina).

### NGS Data Analysis

The biosynthesized DNA was evaluated primarily by error type and its rate, total yield, and average stepwise yield, which were calculated by a Python script with the input of NGS raw data. First, the sequences for all synthetic oligo were extracted from the sequencing reads by a pattern matching the target region (Int‐Extension Part‐PolyA: /AGTGC TACTA GGACG ACTCG AATT (.*?) AAAAA AAAAA/). Subsequently, all extracted sequences were aligned to the target oligonucleotide sequence, and the error types of each synthesis round were divided into three classes, including deletion (Del), insertion (Ins), and substitution (Sub). After classification, the correctly synthesized bases and errors in different locations were counted. Conclusively, the error rate of each type was calculated by *N*_error_ */N*_all_ × 100%, where *N*_error_ is the number of each error and *N*_all_ is the number of all extracted sequences multiplied by the number of synthetic rounds. By the same token, the proportion of correct bases was calculated by *N*_right_ */N*_all_ × 100%, where *N*_right_ is the number of correctly synthesized bases. The full‐length yield was calculated by *S*_right_ */S*_all_ × 100%, where *S*_right_ is the number of correct synthetic sequences and *S*_all_ is the number of all extracted sequences. Average stepwise yield was calculated by (*Y*_total_)^(1/^ *^r^* ^)^, where *Y*_total_ is referred to the full‐length yield and *r* is referred to the number of synthetic rounds. To decode the stored information, a majority vote strategy was applied to all NGS reads to determine the dominant sequence. Majority vote strategy was employed traversing position‐by‐position from the initiator primer as well, to determine the dominant base call and quantify error types. Specifically, each position was sequentially subjected to majority voting to establish its consensus base, followed by error profiling. This analysis was then refined by focusing only on reads that were correct up to the previous position, iterating across all 60 synthesis sites.

### Structural Integrity Evaluation

To rigorously evaluate the structural integrity of the TDN scaffolds following 60 synthesis cycles, complementary PAGE and FRET analyses were employed. Notably, conventional covalent bond cleavage from the interface was deemed unsuitable for objective stability assessment due to its harsh denaturing conditions. Instead, a mild competitive release strategy was implemented using desthiobiotin‐anchored TDN (Table S1, Supporting Information) immobilized on streptavidin‐functionalized PDMS interfaces (modified via standard MPTS/GMBS chemistry). Post‐synthesis, TDN release was achieved through incubation with 1 mm biotin solution (37 °C, 30 min), which competitively displaced the desthiobiotin‐streptavidin interaction. The released products were then analyzed by 8% native PAGE. For FRET‐based evaluation, TDN were assembled with fluorophore‐labeled strands (Cy3 on B‐strand, Cy5 on D‐strand). FRET efficiency measurements before and after synthesis provided quantitative assessment of structural stability through donor‐acceptor distance changes.

### Statistical Analysis

The experimental data were presented as mean ± standard deviation without additional preprocessing. Data visualization included scatter plots and bar graphs for presenting mean values with error bars representing SD. All analyses were performed with *n* = 3 independent replicates per group. Statistical significance was determined using two‐tailed t‐tests and one‐way ANOVA with significance levels denoted as ^*^p <0.05, ^**^p <0.01, ^***^p <0.001, and n.s. for not significant. Image analysis was conducted using ImageJ 1.49 V for quantifying fluorescence intensity and PAGE band densitometry. GraphPad Prism 9.5 was employed for generating scatter plots, bar graphs, heatmaps, and line graphs, as well as for performing linear regression, Michaelis‐Menten fitting, Lineweaver‐Burk plotting, ANOVA, and t‐tests. NGS fasta file processing, including yield and error rate calculations, was performed using Python 3.13.5.

## Conflict of Interest

The authors declare no conflict of interest.

## Author Contributions

K.L. and D.T. contributed equally to this work. K.L. designed the experiments, wrote the original manuscript (lead), and analyzed the data (lead). D.T. conducted the experiments (lead), collected and analyzed the data (supporting), and wrote the original manuscript (supporting). X.L. provided essential reagents (E‐ZaTdT). X.Y. conducted the experiments (supporting). L.L. and Z.J. collected and analyzed the data (supporting). Z.Z. and C.Y. contributed to funding acquisition. Y.T. and H.Z. conceptualized the study, managed project administration, and revised the manuscript. All authors contributed to the manuscript.

## Supporting information



Supporting Information

## Data Availability

The data that support the findings of this study are available in the supplementary material of this article.
