# Supplementary material for: Highly Ordered DNA Framework Interface Enables Efficient Enzymatic Oligonucleotide Synthesis
Source: Adv Sci (Weinh). 2025 Sep 3;12(44):e05868. doi: 10.1002/advs.202505868 (PMC12667552; doi:10.1002/advs.202505868)
Supplement: Supplementary file 1 — Supporting Information [file ADVS-12-e05868-s001.pdf]

WILEY-VCH

Supporting Information

Highly Ordered DNA Framework Interface Enables Efficient Enzymatic Oligonucleotide Synthesis

Kunji Li,[†] Dongbao Tang,[†] Xiaoyun Lu, Xinyao Yang, Luxuan Liu, Zhaoyuan Jia, Zhi Zhu, Yuyu Tan,[*] Huimin Zhang,[*] Chaoyong Yang

Fig. S1. Schematic representation of the surface modification process. PDMS: polydimethylsiloxane; MPTS: mercaptopropyltrimethoxysilane; GMBS: n-γ-maleimidobutyryl-oxysuccinimide ester; NHS: n-hydroxysuccinimide; BSA: bovine serum albumin; SS: single-strand scaffold; DS: double-strand scaffold; TDN: TDN scaffold.

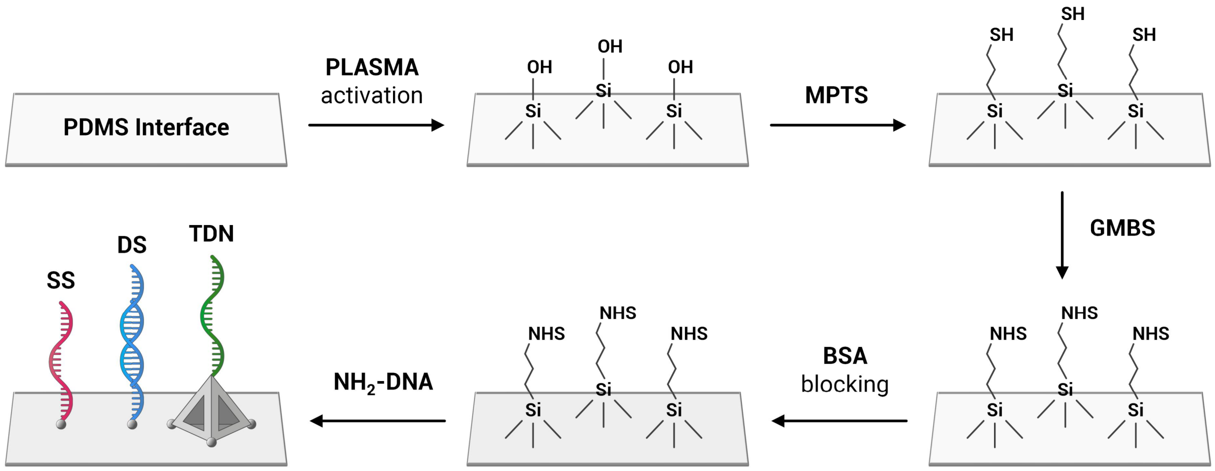

Fig. S2. Fluorescence quantification of modifying the initiator primers on the PDMS interface. The single-stranded DNA (SS-DNA), double-stranded DNA (DS-DNA), and TDN we functionalized the PDMS interface with using the MPTS/GMBS method. To quantify the density of immobilized initiator strands, we hybridized the interface-bound DNA with a complementary FAM-labeled oligonucleotide (FAM-4A-Int') and measured the fluorescence intensity using fluorescence microscopy. Statistical differences between groups were assessed using one-way ANOVA. **p < 0.05, **p < 0.01, ***p < 0.001, n. s. = not significant*. Data are calculated from n = 3 independent experiments and presented as mean ± SD.

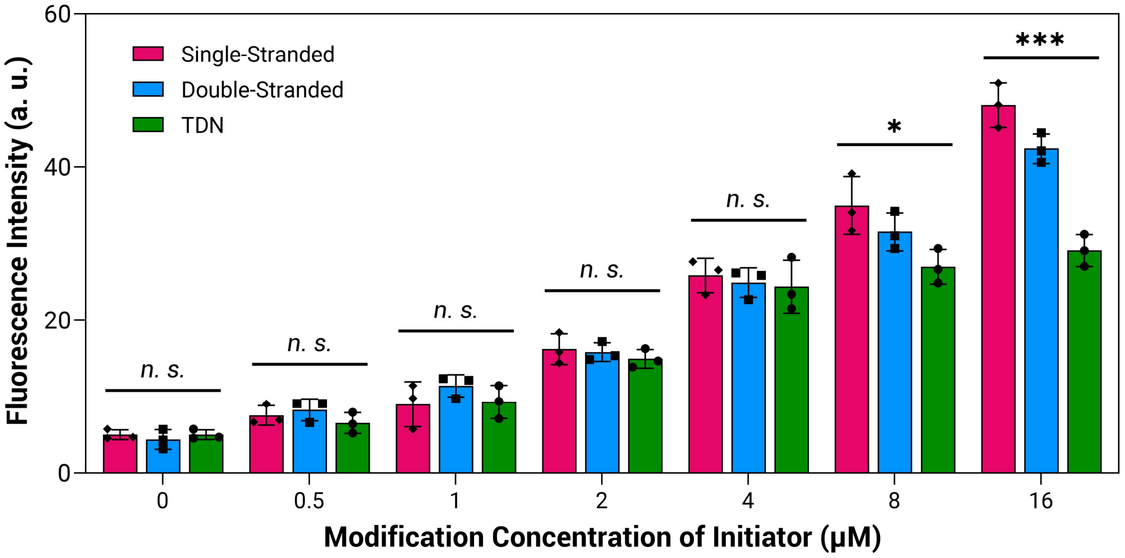

Fig. S3. Characterization of bioluminescence assay. (**A**) Linear correlation between PPi concentration and luminescent intensity in the bioluminescence assay using a multienzyme cascade reaction. (**B**) Recovery analysis of the bioluminescence assay, tested at low (1.88 μM), medium (3.75 μM), and high (7.50 μM) spiked PPi concentrations within the linear range. Data are calculated from n = 3 independent experiments and presented as mean ± SD.

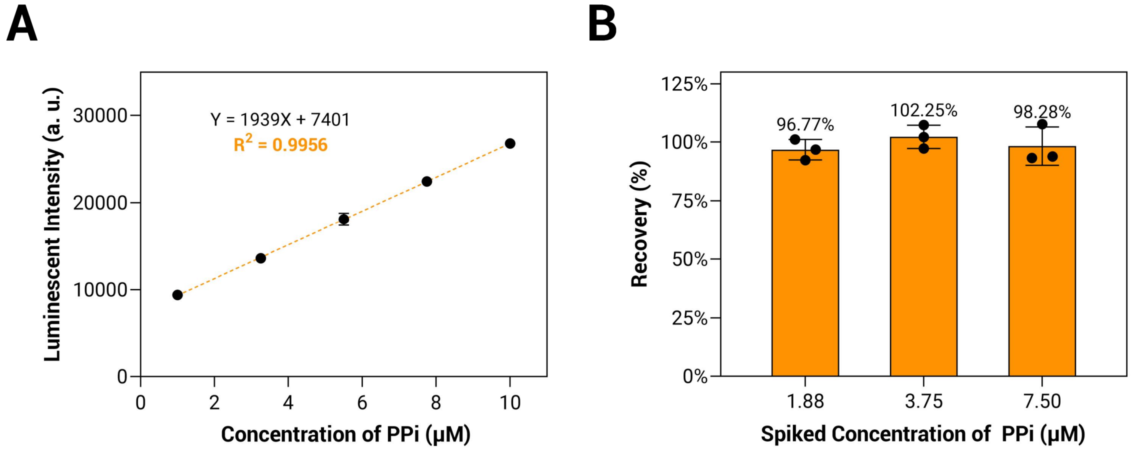

Fig. S4. Characterization of nonspecific confounding effects by TDN scaffold. A comparative analysis of three TDN types composed with different modified strand A termini—phosphate-blocked (TDN-P), hydroxyl-terminated (TDN-OH), and initiator-extended (TDN-Int-OH) groups—was conducted using PPi-based bioluminescence assays. Data are calculated from n = 3 independent experiments and presented as mean ± SD.

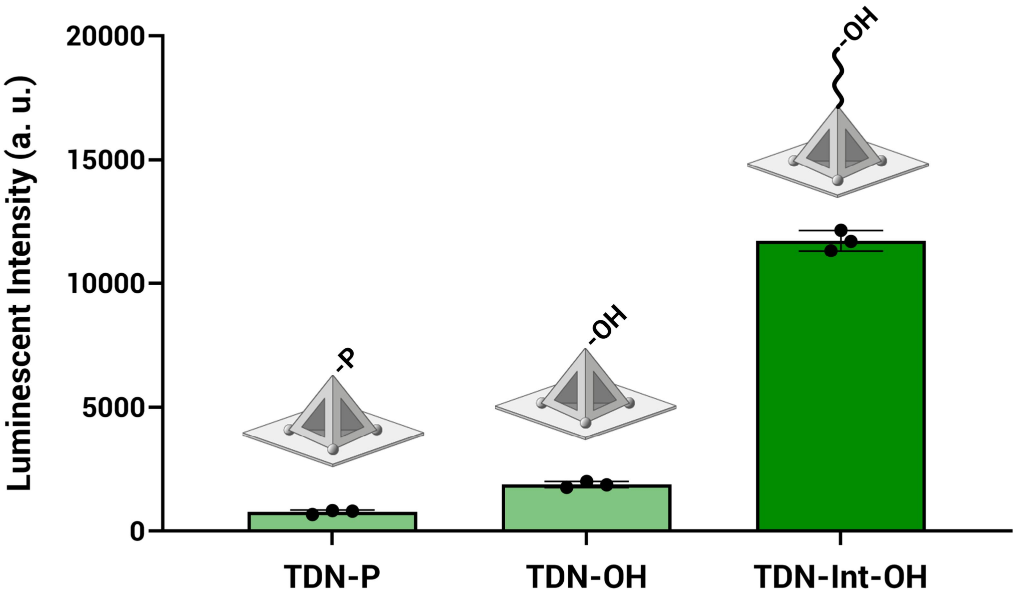

Fig. S5. Optimization of EOS. The optimized experimental parameters including: (**A**) Reaction temperature (20 °C, 30 °C, 40 °C and 50 °C); (**B**) Cobalt ion concentration (0 μM, 0.25 μM, 0.63 μM and 1.25 μM) and (**C**) Surfactant Triton X-100 concentration (0%, 0.005%, 0.01% and 0.02%, v/v). Data are calculated from n = 3 independent experiments and presented as mean ± SD.

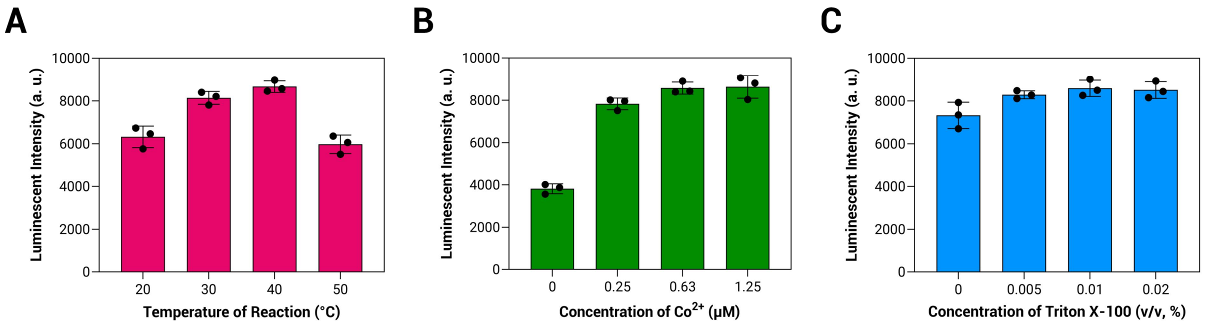

Fig. S6. Comparison of the EOS kinetics for TDN, DS and SS scaffolds. (**A**) Michaelis-Menten curves; (**B**) K_m: kinetic parameter for substrate affinity; (**C**) k_{cat}/K_m: kinetic parameter for catalytic efficiency. Data are calculated from n = 3 independent experiments and presented as mean ± SD.

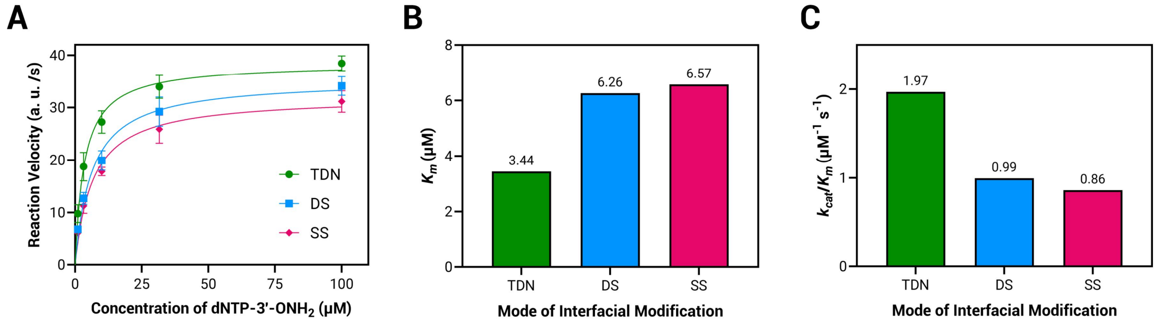

Fig. S7. Comparative analysis of FRET efficiency in EOS kinetics for SS, DS and TDN scaffolds at 1 min (as initial reaction rate) and 10 min (as final product yield) time points. Data are calculated from n = 3 independent experiments and presented as mean ± SD.

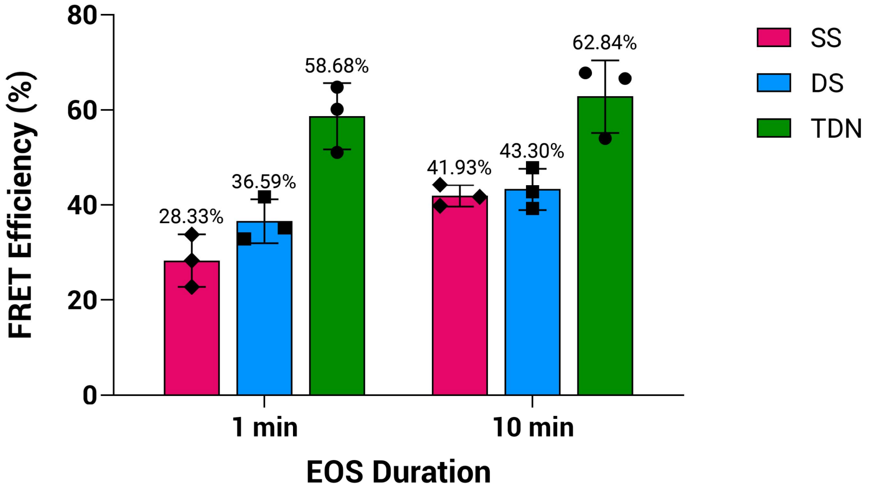

Fig. S8. Comparison of the yield and deletion rate of EOS for 9-base DNA fragment on SS, DS, and TDN scaffolds. (**A**) Schematic representation of initiator primers for EOS on SS, DS, and TDN scaffolds. The comparative parameters include: (**B**) stepwise yield, (**C**) full-length yield, and (**D**) proportion of deletion errors. Data are calculated from n = 3 independent experiments and presented as mean ± SD.

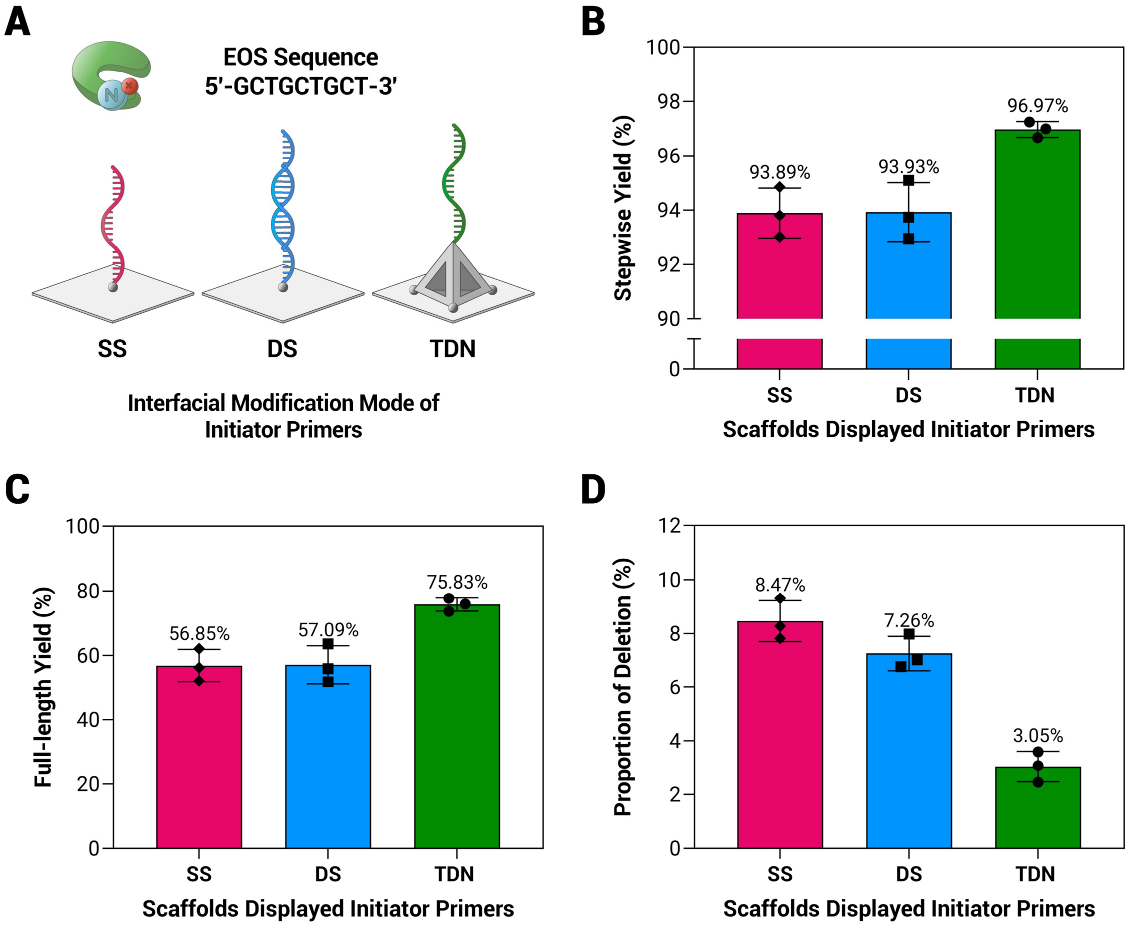

Fig. S9. The top 20 sequences with the highest proportions obtained from NGS analysis.

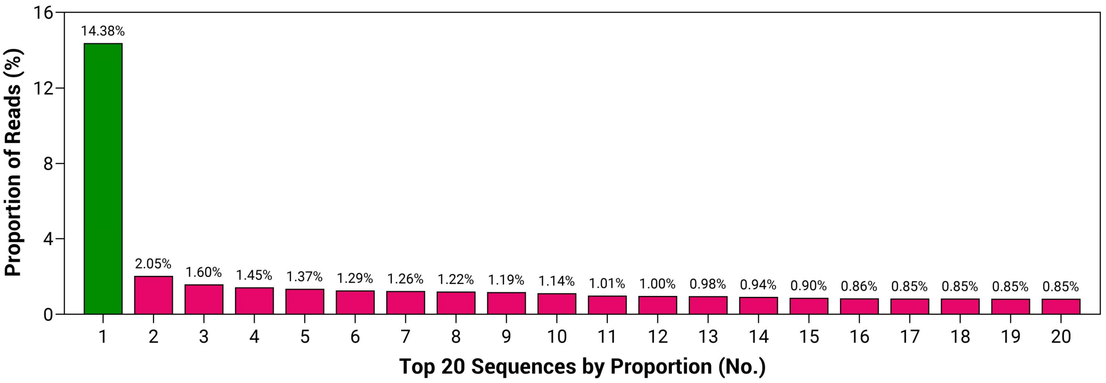

Fig. S10. Error profile analysis across 60 synthesis cycles. (**A**) Full-length error distribution of 60-nt products. (**B**) Position-by-position error distribution using majority-vote traversal. Error profile analysis categorized each position into four types: correct bases (Right), deletions (Del), insertions (Ins), and substitutions (Sub).

A

B

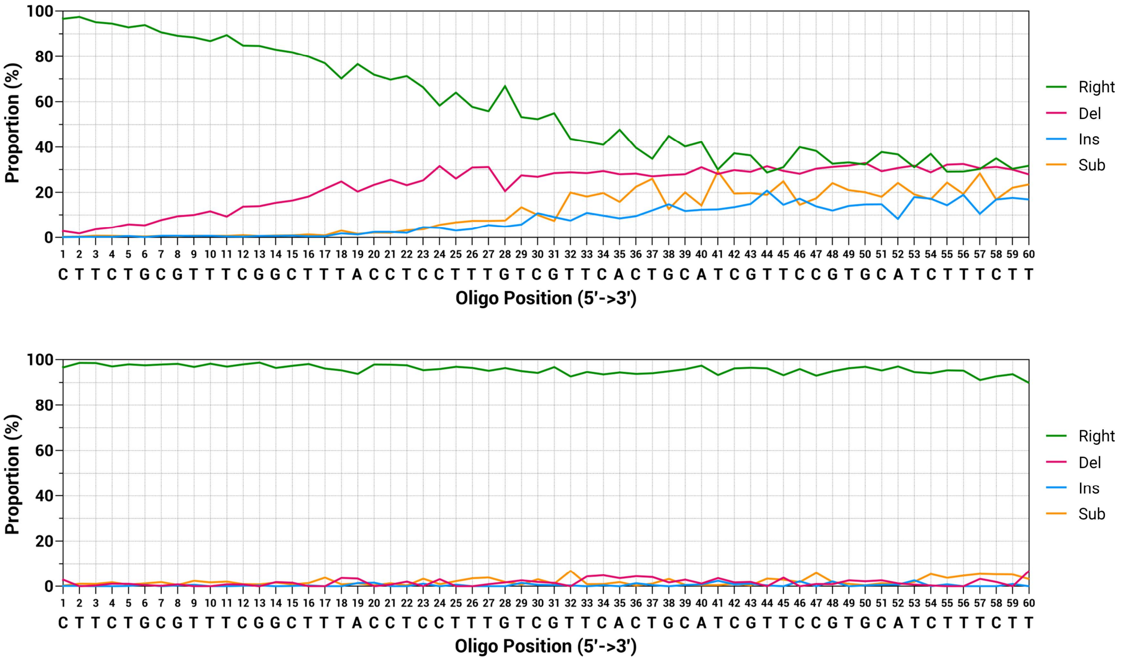

Fig. S11. Structural integrity evaluation of TDN scaffolds after 60 synthesis cycles. (**A**) Native PAGE (8%) analysis of TDN structures before and after synthesis. (**B**) FRET analysis of TDN structures before and after synthesis. Statistical differences between groups were assessed using two-tailed t-test. **p < 0.05, **p < 0.01, ***p < 0.001, n. s. = not significant.* Data are calculated from n = 3 independent experiments and presented as mean ± SD.

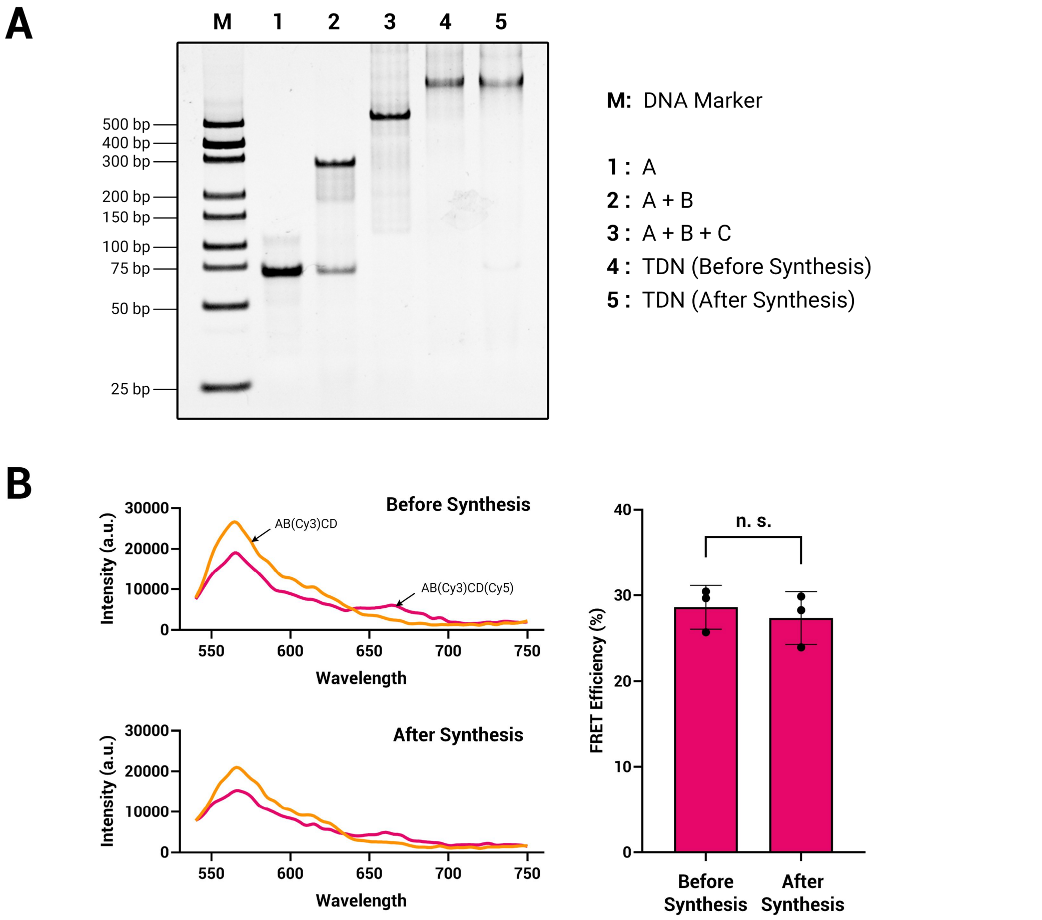

Table S1. DNA sequences used for experiments in this work.

Name	Length	Sequence (5' to 3')	Application
SS-Int	31 nt	NH_2-TTTTTTTTTTTTTACTAGGACGACTCGAATT	Single-stranded scaffold
DS-A-Int	32 nt	AAAAAAAAAAAAAAACTAGGACGACTCGAATT	Double-stranded scaffold
DS-B	13 nt	TTTTTTTTTTTTT-NH_2	
TDN-A-Int	79 nt	ACATTCCTAAGTCTGAAACATTACAGCTTGCTA CACGAGAAGAGCCGCCATAGTA TTTTTT -HS-SH- ACTAGGACGACTCGAATT	TDN scaffold [a]
TDN-A	55 nt	ACATTCCTAAGTCTGAAACATTACAGCTTGCTA CACGAGAAGAGCCGCCATAGTA	
TDN-B	55 nt	TATCACCAGGCAGTTGACAGTGTAGCAAGCTGT AATAGATGCGAGGGTCCAATAC-NH_2	
TDN-C	55 nt	TCAACTGCCTGGTGATAAAACGACACTACGTGG GAATCTACTATGGCGGCTCTTC-NH_2	
TDN-D	55 nt	TTCAGACTTAGGAATGTGCTTCCCACGTAGTGT CGTTTGTATTGGACCCTCGCAT-NH_2	
TDN-A-P	55 nt	ACATTCCTAAGTCTGAAACATTACAGCTTGCTA CACGAGAAGAGCCGCCATAGTA-P	Characterization of nonspecific confounding
Cy3-TDN-B	55 nt	Cy3-TCAACTGCCTGGTGATAAAACGACACTA CGTGGGAATCTACTATGGCGGCTCTTC-NH_2	FRET analysis for TDN structural integrity validation
Cy5-TDN-D	55 nt	Cy5-ACATTCCTAAGTCTGAAACATTACAGCT TGCTACACGAGAAGAGCCGCCATAGTA-P	
TDN-B-^{DT}Biotin	55 nt	TATCACCAGGCAGTTGACAGTGTAGCAAGCTGT AATAGATGCGAGGGTCCAATAC- ^{DT}Biotin	PAGE analysis for TDN structural integrity validation
TDN-C-^{DT}Biotin	55 nt	TCAACTGCCTGGTGATAAAACGACACTACGTGG GAATCTACTATGGCGGCTCTTC- ^{DT}Biotin	
TDN-D-^{DT}Biotin	55 nt	TTCAGACTTAGGAATGTGCTTCCCACGTAGTGT CGTTTGTATTGGACCCTCGCAT- ^{DT}Biotin	
FAM-4A-Int'	22 nt	FAM-AAAAAATTCGAGTCGTCCTAGT	Characterization of interfacial modification
Int	18 nt	ACTAGGACGACTCGAATT	NGS for EOS evaluation
C1	54 nt	GTGACTGGAGTTCAGACGTGTGCTCTTCCGATC TTTTTTTTTTTTTTTTTTTTT	
P5 [b]	94 nt	AATGATACGGCGACCACCGAGATCTACAC NNNNNNNNACACTCTTTCCCTACACGACGCTCT TCCGATCTAGTGCTACTAGGACGACTCGAATT	
P7 [c]	52 nt	CAAGCAGAAGACGGCATACGAGAT NNNNNNNN GTGACTGGAGTTCAGACGTG	

[a] Assembly of TDN scaffold:

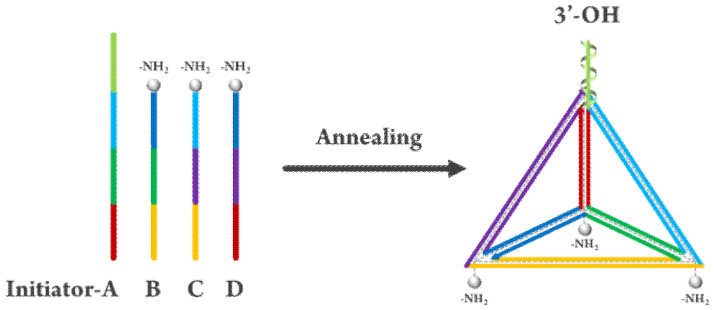

[b] NNNNNNNN in the sequence denotes the 8-base index 5 at the P5 end.

[c] NNNNNNNN in the sequence denotes the 8-base index 7 at the P7 end.

Table S2. DNA sequences synthesized by EOS in this work.

Name	Length	Sequence (5' to 3')	Remarks
9-nt fragment	9 nt	GCTGCTGCT	
Homopolymers-C	3 nt	CCC	
Homopolymers-T	6 nt	TTTTTT	
High GC-80%	10 nt	CGCGTCGCGA	
Low GC-20%	10 nt	ATATGATATC	
Hairpin Structure	16 nt	CGAGCTAGTCAGCTCG	Free energy ΔG = -7.10 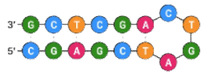
60-nt fragment	60 nt	CTTCTGCGTTTCGGCTTTACCTCCTT TGTCGTTCACTGCATCGTTCCGTGCA TCTTTCTT	Information storage

Table S3. Encoding table for "陈嘉庚 TanKahKee" (15 bytes) within a 60-nt oligonucleotide sequence.

Number	Character	UTF Code	Binary Code	DNA Sequence
1	陈	u9648	1001011001001000	CTTCTGCG
2	嘉	u5609	0101011000001001	TTTCGGCT
3	庚	u5e9a	0101111010011010	TTACCTCC
4	T	u0054	01010100	TTTG
5	a	u0061	01100001	TCGT
6	n	u006e	01101110	TCAC
7	K	u004b	01001011	TGCA
8	a	u0061	01100001	TCGT
9	h	u0068	01101000	TCCG
10	K	u004b	01001011	TGCA
11	e	u0065	01100101	TCTT
12	e	u0065	01100101	TCTT
